# Preeclampsia, antihypertensive medication use in pregnancy and risk of childhood cancer in offspring

**DOI:** 10.1007/s10552-023-01745-4

**Published:** 2023-08-03

**Authors:** Lexie Askins, Helen T. Orimoloye, Chuanjie Deng, Johnni Hansen, Jorn Olsen, Beate Ritz, Carla Janzen, Julia E. Heck

**Affiliations:** 1 Department of Epidemiology, Fielding School of Public Health, University of California (UCLA), Box 951772, Los Angeles, CA 90095-1772 USA; 2 Present Address: College of Health and Public Service, University of North Texas, 1155 Union Circle #311340, Denton, TX 76203-5017 USA; 3 Danish Cancer Society Research Center, Strandboulevarden 49, 2100 Copenhagen, Denmark; 4 Department of Clinical Epidemiology, Aarhus University, Olof Palmes Allé 43-45, 8200 Aarhus N, Denmark; 5 Department of Obstetrics and Gynecology, University of California, Box 951740, Los Angeles, CA USA

**Keywords:** Preeclampsia, Childhood cancer epidemiology, Hypertension, Antihypertensives, Diuretics

## Abstract

**Purpose:**

Preeclampsia is a serious pregnancy complication that presents a significant risk to both the mother and the fetus. Preeclampsia and medications associated with its treatment are potentially linked to increased childhood cancer risk. Therefore, we examined the association between preeclampsia, antihypertensive medications, and childhood cancer in offspring.

**Methods:**

Cases (n = 6,420) and controls (n = 160,484) were obtained from Danish national registries. We performed conditional logistic regression analyses to estimate the association between preeclampsia and childhood cancer risk, and examined the effects of antihypertensive medication use in pregnancy in relation to childhood cancer risk in the offspring with adjustment for relevant covariates.

**Results:**

We observed an increased risk of acute lymphoblastic leukemia (ALL) among those whose mothers had preeclampsia (OR = 1.36, 95% CI 1.03, 1.79), especially for severe preeclampsia (OR = 2.36, 95% CI 1.37, 4.08). We also estimated an increased cancer risk in children born to mothers who were prescribed diuretics during pregnancy [OR = 2.09, 95% confidence interval (CI) 1.39, 3.14]. Intake of other antihypertensive medications was not associated with childhood cancer (OR = 0.78, 95% CI 0.50, 1.23). Among women who did not take diuretics in pregnancy, preeclampsia was associated with neuroblastoma (OR = 2.22, 95% CI 1.08, 4.55).

**Conclusion:**

Our findings suggested an increased risk for certain types of cancer in the offspring of mothers with preeclampsia and an increased risk of cancer with diuretic intake during pregnancy.

**Supplementary Information:**

The online version contains supplementary material available at 10.1007/s10552-023-01745-4.

## Introduction

Preeclampsia, also referred to as toxemia, is hypertension during pregnancy that may damage the liver and kidneys and is accompanied by proteinuria with other symptoms such as low platelet count or vision disturbance. This condition occurs in 2–8% of all pregnancies globally [1]. Preeclampsia is a two-stage disorder, initiated by placental perfusion and followed by the maternal systemic syndrome [2, 3]. Reduced placental perfusion is due to a failure of spiral arteries to supply blood to the placenta i.e. to undergo the necessary changes in order to allow for normal blood flow. The maternal syndrome of preeclampsia refers to a sequence of events resulting from reduced placental perfusion which leads to hypertension, proteinuria, and other symptoms typically observed. Preeclampsia can adversely affect the fetus in a number of ways, including through low birth weight and preterm birth—associations that are in part explained by induced preterm delivery—yet preeclampsia is also associated with increased intrauterine growth restriction [2, 3].

Cancer is a primary cause of disease-related mortality among children in developed countries, yet its causes are less amply understood in comparison to adult cancers. Adverse perinatal outcomes related to preeclampsia (low birth weight, preterm delivery) are linked to childhood cancer [4]. Some studies have reported positive associations between preeclampsia and overall childhood cancer [5, 6], soft tissue sarcoma [7], and most consistently, hepatoblastoma [4, 8–10]. Research on preeclampsia and other cancer types have produced mixed results, with some studies suggesting an increased risk for neuroblastoma and acute lymphoblastic leukemia (ALL) [8, 11–17]. Overall, due to the rarity of childhood cancer, epidemiological evidence on the relationship between preeclampsia and the risk of childhood cancer is insufficient in both the number of studies and sample size. Registry-based studies examining the association between preeclampsia and childhood cancers are especially limited [8], and even fewer studies include rarer cancer types.

Increases in offspring cancer may be due to medications used to treat preeclampsia rather than the condition itself. Guidelines have suggested several options for the treatment of preeclampsia and pregnancy-related hypertension including medications such as aspirin, diuretics or beta-blockers, and/or to induce early delivery [18]. Several studies reported an increased childhood cancer risk with antihypertensive medication use [19–26] yet some of them grouped all types of medication together [19, 24], making it difficult to ascertain which medications were implicated. Further, the use of medication was often self-reported and thereby prone to recall or report bias. The biological mechanism through which medications used to treat preeclampsia may increase the risk of childhood cancer is not clear, but some of these medications have been found to be associated with fetal growth restriction and further plasma reduction [18].

Therefore, we conducted this population-based matched case–control study to evaluate the association between preeclampsia and/or the treatment of maternal hypertension and childhood cancer risk using national registries in Denmark.

## Material and methods

As described previously [27], all primary cancer cases diagnosed between 1977–2016 in children < 20 years (births 1977–2013) were ascertained from the Danish Cancer Registry. Specific cancer subtypes were grouped according to the International Childhood Cancer Classification corresponding based on ICD-O site, histology, and behavior. Eligible controls, alive and cancer-free at the time of the corresponding case’s diagnosis, were randomly selected from the Central Population Register and matched to cases by sex and birth date with a matching ratio of 25:1. Only children born in Denmark were included in the study. Demographic information came from the Central Population Register. The study used existing sources, with no informed consent required.

Participants’ gestational and medical information was obtained from the Medical Births Registry and the National Patient Registry [28, 29]. The Medical Births Registry contains data on all live births by women with permanent residence in Denmark from 1973, including information on home births, preterm births, birthweight, and gestational age. Gestational age was based on the date of last menstrual period; when it was missing we estimated gestational age as previously described [30]. The National Patient Registry includes information on all hospital admissions since 1977 and outpatient visits since 1994. Diagnostic information was based on the Danish version of the International Classification of Diseases (ICD), Eighth Revision (ICD-8) up until 1993 and subsequently on the tenth revision (ICD-10); the ICD codes used to identify preeclampsia [including eclampsia and hemolysis, elevated liver enzymes and low platelets (HELLP) syndrome] and hypertension were shown in Supplemental Table S1. Hypertension was defined as any diagnosis before or during the index pregnancy. Gestational hypertension, defined as hypertension occurring after the 20th week of pregnancy, accounted for a small proportion of all hypertension in our sample (9.1%), and we grouped these diagnoses with chronic hypertension. Although hypertension may be independently associated with childhood cancer [16, 26, 31], it was too rare in our sample to independently examine this exposure.

Preeclampsia status was further stratified into categories labeled as mild, severe, or unspecified, using ICD-codes previously identified [5]. Preeclampsia was categorized as mild (*ICD-8*: 637.03; *ICD-10*: O14.0), severe (*ICD-8*: 637.04; *ICD-10*: O14.1), or unspecified (*ICD-8*: 637.09; *ICD-10*: O149), and eclampsia cases were also identified (*ICD-8*: 637.1; *ICD-10*: O15). Mild preeclampsia is normally defined as the presence of proteinuria and hypertension in a pregnant woman after 20 weeks gestation. In contrast, severe preeclampsia is the presence of greater than 300 g proteinuria and hypertension with at least one additional symptom, either headache or blurring of vision. However, during the earlier time period (≤ 1993), the severity of preeclampsia (mild vs. severe) was less commonly identified than in later years, due to the lack of specificity in ICD-8 coding. In our data, “unspecified” preeclampsia diagnoses accounted for 51% of preeclampsia diagnoses until 1993, and 9% from 1994 onwards. However, among the preeclampsia cases where the severity could be identified, 84.6% were mild ≤ 1993 and 78.2% were mild after 1993; therefore, it is not clear if the “unspecified” preeclampsia diagnoses ≤ 1993 may have included a larger proportion of severe preeclampsia.

We ran this analysis in both the 1977–2013 sample and the Pharmaceutical Register (1995–2014) sample (as described below), but we only reported the results from the 1977–2013 sample because they were better powered. Each category of preeclampsia was recoded as a binary variable and put in the models separately.

We performed conditional logistic regression to evaluate associations of preeclampsia and antihypertensive medication use in pregnancy with the risk of overall childhood cancer as well as different cancer subtypes. Selection of covariates including potential confounders and risk factors was based on the literature [4, 11–13, 23–25, 27, 31–33]. We adjusted our models for maternal age at delivery, birth order (first born/later), mother’s place of birth (Denmark/Other European countries or North America/Other), urban/small town/rural residence, maternal diagnosis of hypertension prior to pregnancy, rheumatoid arthritis before pregnancy, and maternal ever diagnosis of atopic conditions and epilepsy. Maternal place of birth was used as a proxy for ethnic group, as previously described [27]. We considered additional adjustment for other covariates that have been previously suggested as risk factors for childhood cancer, including multiple births, parity, father’s place of birth, as well as maternal chronic conditions that have been previously linked to cancers in children including: diabetes, anemia, body mass index (BMI), and smoking status, but adding them to models did not change results by 10% so they were not included in the final model [34]. BMI is a risk factor for preeclampsia [18], but we were not able to adjust for it in our main analysis because it was only collected during part of the study period (2004+). In sensitivity analyses, we conducted a subgroup analysis for use of antihypertensive medication and risk of cancer in offspring in which we adjusted for BMI in addition to the previously listed covariates. Due to the relatively small number of cases and controls included in this analysis, it was not shown in our main results. Also, we restricted the population to mothers without diuretics use and then modeled childhood cancer risk against preeclampsia.

We conducted analyses in two samples: the first relied on data from the National Patient Register (1977–2013), which allowed for the greatest statistical power due to the coverage of a longer time period. Then, to determine whether an increased risk might be due to medications used to treat hypertension as part of the preeclampsia syndrome, we additionally utilized the National Prescription Register, which includes information on every prescription filled at a pharmacy in Denmark but is only available for the latter part of our study period (births 1995–2014). We estimated odds ratios and interpreted them as risk ratios because of our rare outcomes.

### 1977–2013 Sample and sensitivity analysis

This sample consisted of 6,420 cases and 160,484 matched controls. The National Patient Register included ICD-8 coding from 1977 to 1993, after which it began to use ICD-10**.** Hence, we conducted sensitivity analyses based on two birth year periods (1977–1993 and 1994+), however, results were similar, so we report overall results for the entire time period. In order to compare results to other studies that did not distinguish between preeclampsia and chronic hypertension, we conducted sensitivity analyses combining these diagnoses. We additionally conducted a mediation analysis to determine whether any association was mediated through the relation with congenital anomalies.

### National Prescription Registry Sample (births 1995–2014) and data analysis

As per Danish national regulations, National Prescription Register data are kept on the Statistics Denmark server [35]. Therefore, the analysis that included these medications was done separately with different controls ascertained, although they were sampled after the same principles as above (matched by sex and birth date) and randomly selected for this analysis. Along with the National Prescription Register, this sample included all registries listed above. This analysis included 2,521 cases (diagnosed 1995–2016) and 63,025 controls. Medications were identified by the Anatomical Therapeutic Chemical (ATC) codes (Supplemental Table S1), and medication use was recoded as a binary variable (yes/no) for individual medicines. Because diuretics use was previously reported to be associated with an increased childhood cancer risk [19, 21, 22], we further stratified by types of antihypertensive medications, as statistical power allowed. We utilized conditional logistic regression to estimate the association between childhood cancer and maternal use of antihypertensive medications, adjusting for the same covariates as used in the models for preeclampsia and childhood cancer risk. We only presented results for cancer types with at least five exposed cases.

## Results

The demographic distribution of mothers of cases was similar to that of mothers of controls in both samples (Table 1). In the 1977–2013 sample, the prevalence of preeclampsia in mothers of cases was 243 (3.8%) and 5490 (3.4%) in mothers of controls. The prevalence of hypertension before pregnancy in mothers of cases was 15 (0.2%) and 287 (0.2%) in mothers of controls. In the 1995–2014 sample, the prevalence of diuretic use in pregnancy among mothers of cases was 26 (1%) and 306 (0.5%) in mothers of controls.

**Table 1:** Participants’ demographic characteristics by cancer case and control status

Characteristics	1977–2013 Sample			National Prescription Register Sample (1995–2014)	
	Case	Control		Case	Control
	(N = 6,420)	(N = 160,484)		(N = 2,521)	(N = 63,025)
	Mean (SD) or N (%)	Mean (SD) or N (%)		Mean (SD) or N (%)	Mean (SD) or N (%)
Maternal age at time of index pregnancy (years; mean)	28.5 (5.0)	28.4 (5.0)		29.8 (4.8)	29.9 (4.8)
Paternal age at time of index pregnancy (years; mean)	31.2 (5.8)	31.1 (5.8)		32.4 (5.7)	32.5 (5.8)
First born child (N)	2,828 (44.0)	68,852 (42.9)		1,074 (42.6)	25,405 (40.3)
Mother’s place of birth (N)					
Denmark	5,853 (91.3)	146,777 (91.6)		2,205 (88.0)	55,444 (88.1)
Other Europe & N. America	217 (3.4)	5523 (3.5)		111 (4.4)	2735 (4.3)
Other	338 (5.3)	7893 (4.9)		189 (7.5)	4747 (7.5)
Missing	12	291		16	99
Urban or rural residence					
Urban	2,114 (32.9)	50,981 (31.8)		896 (36.1)	22,054 (35.0)
Small towns	1,808 (28.2)	46,632 (29.1)		677 (27.3)	17,672 (28.0)
Rural	2,498 (38.9)	62,871 (39.2)		910 (36.6)	23,299 (37.0)
Missing	0	0		38	0
Hypertension (before pregnancy)	15 (0.2)	287 (0.2)		14 (0.6)	203 (0.3)
Atopic conditions (lifetime)	385 (6.0)	8712 (5.4)		167 (6.6)	3870 (6.1)
Rheumatoid arthritis (before pregnancy)	17 (0.3)	143 (0.1)		15 (0.6)	104 (0.2)
Epilepsy (lifetime)	148 (2.3)	2978 (1.9)		63 (2.5)	1220 (1.9)
Pre-pregnancy BMI *					
18.4 or less	33 (4.1)	815 (4.1)		37 (4.2)	887 (4.2)
18.5–25	496 (61.0)	12,775 (64.2)		528 (60.6)	13,592 (63.6)
25 +	284 (34.9)	6309 (31.7)		307 (35.2)	6876 (32.2)
Missing	197	5342		186	5095
Smoking status at the first midwife consultation**					
Yes	818 (23.9)	20,753 (24.2)		487 (19.3)	12,452 (19.8)
No	2,601 (76.1)	65,060 (75.8)		2034 (80.7)	50,573 (80.2)
Missing	187	4321		0	0
Child's Birth Weight					
Very low (< 1,500 g)	48 (0.7)	944 (0.6)		21 (0.8)	416 (0.7)
Low (1,500–2,500 g)	367 (5.8)	9,252 (5.8)		111 (4.4)	2,718 (4.3)
Normal (2,501–4,000 g)	5,397 (84.5)	137,499 (86.0)		1,811 (72.4)	47,289 (75.6)
High (> 4,000 g)	575 (9.0)	12,138 (7.6)		556 (22.2)	12,153 (19.4)
Missing	33	651		22	449
Gestational Weeks					
1993 or earlier (mean, SD)	39.5 (1.8)	39.6 (1.8)			
Very preterm (< 33 wks)	29 (0.8)	748 (0.8)			
Preterm (33—36 wks)	157 (4.4)	3,990 (4.5)			
Term (37- 42 wks)	3,366 (94.0)	83,840 (93.7)			
Late (43 +)	29 (0.8)	947 (1.1)			
1994+ (mean, SD)	39.3 (2.1)	39.3 (2.0)	1995–2014	39.3 (2.0)	39.2 (2.1)
Very preterm (< 33 wks)	41 (1.4)	828 (1.2)		36 (1.4)	756 (1.2)
Preterm (33–36 wks)	157 (5.5)	3,607 (5.1)		149 (5.9)	3,294 (5.2)
Term (37- 42 wks)	2,637 (92.9)	66,335 (93.5)		2,333 (92.5)	58,901 (93.5)
Late (43+)	4 (0.1)	189 (0.3)		3 (0.2)	74 (0.1)

*Pre-pregnancy BMI was collected from 2003 to 2013

**Smoking status at the first midwife consultation was collected from 1991 to 2013

### 1977–2013 Sample

Before 1994, the mean gestational weeks at delivery was 39.2 for pregnancies complicated by preeclampsia/eclampsia, and in the later time period, the mean number of weeks reached for preeclamptic pregnancies was 38.0. Before 1994, 95.0% of all pregnancies surpassed full-term status (37 weeks), versus 93.7% of all pregnancies in the later years.

**Fig. 1:**
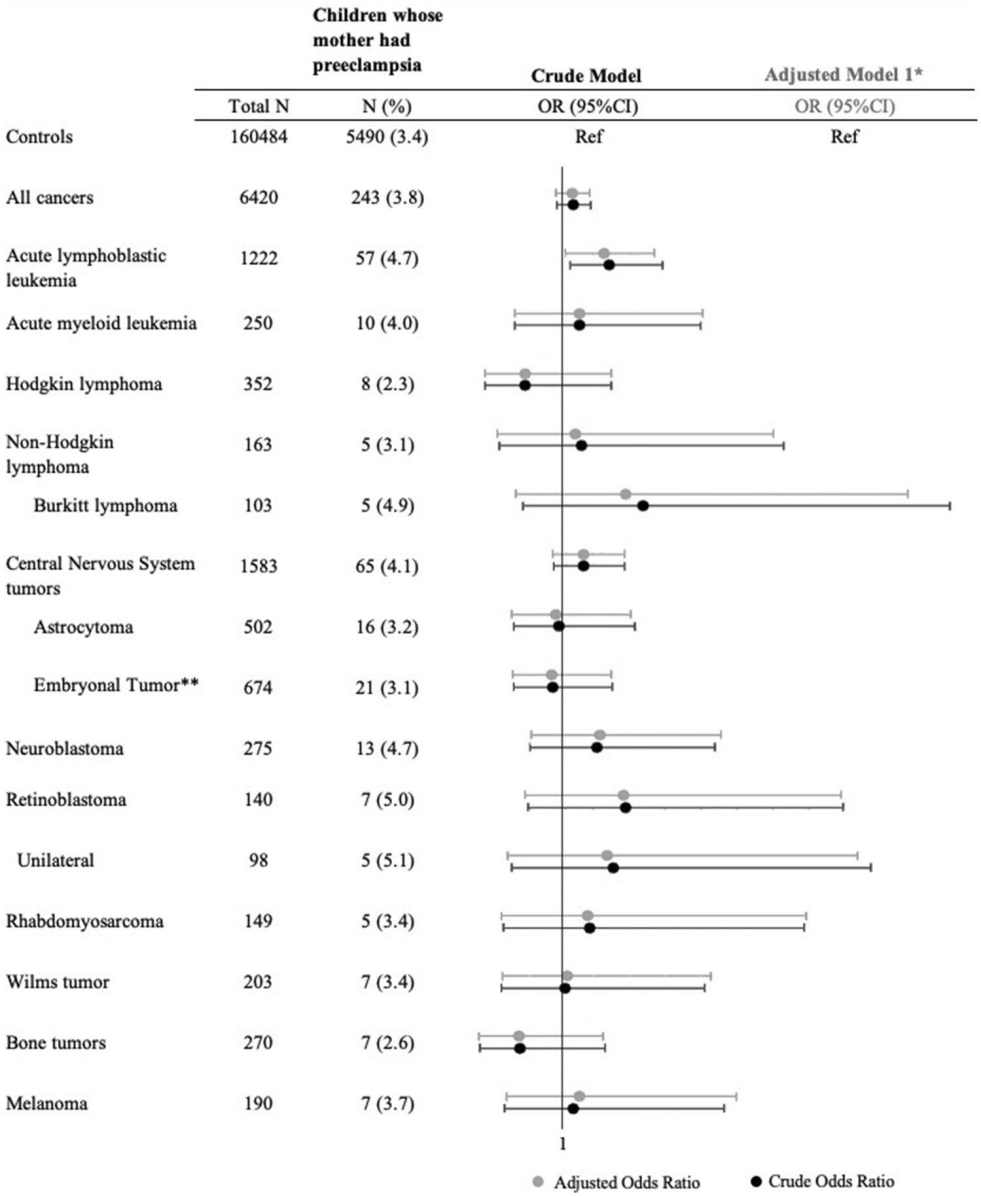
Association between maternal preeclampsia during pregnancy and risk for cancer in offspring, Denmark (1977–2013 Sample)

Using multivariate analysis, we observed an elevated point estimate for the association between preeclampsia and all childhood cancers combined, as well as a positive association between preeclampsia and ALL (OR = 1.36, 95% CI 1.03, 1.79) (Fig. 1). The risk of ALL was higher in the early (before 1994) study period compared to the later (1994+) study period (Table 2). When we stratified by severity of preeclampsia, the risk of ALL was higher when preeclampsia was reported as severe or unspecified, (OR = 2.36, 95% CI 1.37, 4.08) compared to mild.

**Table 2:** Risk of selected cancer types among children born to mothers with a preeclampsia diagnosis, stratified by birth year, preeclampsia severity (National Patient Register Sample; births 1977–2013)

	Births in the year 1993 or earlier				Births after the year 1993			
	Total N	N (%)	Crude Model	Adjusted Model 1*	Total N	N (%)	Crude Model	Adjusted Model 1*
			OR (95% CI)	OR (95% CI)			OR (95% CI)	OR (95% CI)
Controls	89,525	3463 (3.9)	Ref	Ref	70,959	2027 (2.8)	Ref	Ref
All cancers	3,581	157 (4.4)	1.13 (0.96, 1.33)	1.11 (0.94, 1.31)	2,839	86 (3.0)	1.05 (0.84, 1.31)	1.04 (0.83, 1.30)
Acute Lymphoblastic Leukemia	634	35 (5.5)	1.51 (1.06, 2.15)	1.47 (1.03, 2.10)	588	22 (3.7)	1.28 (0.83, 1.99)	1.20 (0.77, 1.87)
Central Nervous System Tumors	895	46 (5.1)	1.32 (0.97, 1.80)	1.32 (0.97, 1.79)	688	19 (2.7)	0.97 (0.61, 1.55)	0.96 (0.60, 1.53)
Neuroblastoma	141	< 5	–	–	134	10 (7.5)	2.36 (1.20, 4.65)	2.27 (1.14, 4.52)

	Total N	Mild preeclampsia			Severe preeclampsia			Unspecified preeclampsia		
			Crude Model	Adjusted Model 1 *		Crude Model	Adjusted Model 1 *		Crude Model	Adjusted Model 1 *
		N (%)	OR (95% CI)	OR (95% CI)	N (%)	OR (95% CI)	OR (95% CI)	N (%)	OR (95% CI)	OR (95% CI)
Controls	160,484	3171 (2.0)	Ref	Ref	720 (0.5)	Ref	Ref	1530 (1.0)	Ref	Ref
All cancers	6,420	127 (2.0)	1.01 (0.84, 1.21)	1.00 (0.83, 1.20)	33 (0.5)	1.12 (0.79, 1.60)	1.11 (0.78, 1.58)	82 (1.3)	1.31 (1.04, 1.64)	1.29 (1.03, 1.61)
Acute Lymphoblastic Leukemia	1,222	27 (2.2)	1.18 (0.80, 1.75)	1.13 (0.77, 1.68)	15 (1.3)	2.47 (1.43, 4.25)	2.36 (1.37, 4.08)	15 (1.3)	1.38 (0.82, 2.35)	1.33 (0.78, 2.25)
Central Nervous System Tumors	1,583	30 (1.9)	0.95 (0.66, 1.38)	0.95 (0.66, 1.38)	9 (0.6)	1.33 (0.68, 2.62)	1.33 (0.68, 2.62)	25 (1.6)	1.61 (1.07, 2.44)	1.61 (1.07, 2.44)
–Astrocytoma	502	7 (1.4)	0.70 (0.33, 1.50)	0.67 (0.31, 1.44)	< 5	–	–	7 (1.4)	1.59 (0.73, 3.45)	1.59 (0.73, 3.47)
–Intracranial and Intraspinal Embryonal Tumor	674	9 (1.3)	0.74 (0.38, 1.43)	0.73 (0.38, 1.43)	< 5	–	–	9 (1.3)	1.30 (0.66, 2.58)	1.30 (0.66, 2.58)
Neuroblastoma	275	6 (2.2)	1.08 (0.47, 2.48)	1.09 (0.48, 2.51)	< 5	–	–	6 (2.2)	1.96 (0.83, 4.61)	2.02 (0.85, 4.79)

*Model adjusted for maternal age at delivery, birth order (first/later born), mother’s place of birth, urban or rural deliveries, hypertension before index pregnancy, atopic conditions (lifetime), rheumatoid arthritis before pregnancy, epilepsy (lifetime)

We observed an increased risk for central nervous system (CNS) tumors in the earlier study period. The estimations for risk of CNS tumors were higher when preeclampsia was reported as unspecified or severe, although the results for CNS tumors and severe preeclampsia were imprecise. Overall, we found decreased point estimates in the later time period for all cancers combined, ALL, and CNS tumors compared to the earlier time period; however, the confidence intervals for the earlier and later time periods overlapped.

Although results were imprecise, we also observed elevated point estimates for preeclampsia and the risk of the following cancer types: Burkitt lymphoma, neuroblastoma, and retinoblastoma (Fig. 1, Supplemental Table S2).

In sensitivity analyses adjusting for maternal BMI, which included 872 cases and 21,355 controls, power was limited to examine most cancer types. We observed an increased association between preeclampsia and neuroblastoma (OR = 3.05, 95% CI [1.10, 8.42]; results for other cancers not shown). In the sensitivity analysis that combined preeclampsia and hypertension, results were similar to overall findings for all cancers combined, ALL, CNS tumors, and neuroblastoma. (Supplemental Table S3). The combined results for rhabdomyosarcoma and unilateral retinoblastoma were slightly elevated in this sensitivity analysis, while the estimate for Burkitt lymphoma was weaker.

In the mediation analysis, the OR for the total effect was 1.02 (95% CI [0.78, 1.27], whereas the OR for controlled direct effect accounting for congenital anomalies was 1.02 (95% CI [0.77, 1.26]). The percentage mediated by congenital anomalies was 29.5%. In conclusion, part of the association between preeclampsia and childhood cancer could be explained by the mediation from congenital anomalies.

### National Prescription Register Sample (births 1995–2014)

In our analysis of antihypertensive medications in the National Prescription Register sample, we observed elevated risks of all cancers combined and ALL for use of any antihypertensive medications (Table 3). We found a two-fold risk of all cancers combined in the offspring of women who had ever taken diuretics in pregnancy (OR = 2.09, 95% CI [1.39, 3.14]). Results for diuretic use in pregnancy and risk of both ALL and CNS tumors included elevated point estimates, but both confidence intervals overlapped the null. Among those who took diuretics (n = 332): 4.2% (n = 14) received a diagnosis of only pre-pregnancy hypertension, 11.1% (n = 37) received a diagnosis of only preeclampsia, and 0.6% (n = 2) received a diagnosis of both pre-pregnancy hypertension and preeclampsia. For women who took antihypertensive medications other than diuretics, there was no increase in cancer risk.

**Table 3:** Association between antihypertensive medication use during pregnancy and risk of offspring cancers, (National Prescription Register Sample; births $\ge$ 1995)

	Prevalence of medication use	Crude model	Adjusted model 1*
	N (%)	OR (95% CI)	OR (95% CI)
Any Antihypertensive Medications			
Controls	936 (1.5)	Ref	Ref
All Cancers	47 (1.9)	1.26 (0.94, 1.69)	1.22 (0.90, 1.67)
Acute Lymphoblastic Leukemia	14 (2.5)	1.63 (0.94, 2.82)	1.42 (0.79, 2.55)
Central Nervous System Tumors	9 (1.5)	1.09 (0.57, 2.13)	1.19 (0.59, 2.37)
Diuretics			
Controls	306 (0.5)	Ref	Ref
All Cancers	26 (1.0)	2.13 (1.43, 3.19)	2.09 (1.39, 3.14)
Acute Lymphoblastic Leukemia	7 (1.3)	2.30 (1.05, 5.01)	2.01 (0.91, 4.46)
Central Nervous System Tumors	5 (0.8)	2.02 (0.81, 5.04)	2.18 (0.87, 5.47)
Antihypertension Medications other than Diuretics			
Controls	630 (1.0)	Ref	Ref
All Cancers	21 (0.8)	0.83 (0.54, 1.29)	0.77 (0.49, 1.21)
Acute Lymphoblastic Leukemia	7 (1.3)	1.25 (0.58, 2.69)	1.04 (0.46, 2.33)

*The comparison group consists of offspring of women who did not use any type of antihypertension medication during pregnancy

*Model adjusted for maternal age at delivery, firstborn child, mother's place of birth, urban or rural deliveries, hypertension before index pregnancy, atopic conditions (lifetime), rheumatoid arthritis before pregnancy, epilepsy (lifetime), preeclampsia

When we examined childhood cancer risk among women who had either a diagnosis of hypertension or preeclampsia but did not take diuretics, there was no increased risk of all cancers (combined) but an increase in neuroblastoma remained (Table 4).

**Table 4:** Among women who had preeclampsia but did not take diuretics in pregnancy, the risk of offspring cancer (National Prescription Register Sample; births $\ge$ 1995)

	Children whose mother had preeclampsia	Crude model	Adjusted model 1*
	N (%)	OR (95%CI)	OR (95%CI)
Controls	1710 (2.7)	Ref	Ref
All Cancers	73 (2.9)	1.08 (0.85, 1.36)	1.01 (0.79, 1.29)
Acute Lymphoblastic Leukemia	19 (3.5)	1.27 (0.80, 2.01)	1.23 (0.77, 1.95)
Neuroblastoma	8 (6.0)	2.26 (1.10, 4.62)	2.22 (1.08, 4.55)
Central Nervous System Tumors	18 (3.1)	1.12 (0.70, 1.80)	1.02 (0.62, 1.68)

^*^Model adjusted for maternal age at delivery, first born child, mother’s place of birth, urban or rural deliveries, hypertension before index pregnancy, atopic conditions (lifetime), rheumatoid arthritis before pregnancy, epilepsy (lifetime)

## Discussion

In this population-based matched case–control study which relied on medical records, we observed associations between diuretic intake in pregnancy and risk of childhood cancer, and an elevated point estimate for the association between diuretic use and ALL. In addition, we observed increases in neuroblastoma among mothers with preeclampsia, even when they did not take diuretics. Our population-based study is large for a study of childhood cancer and allowed us to assess associations for multiple cancer subtypes; however, confidence intervals were wide for some types, producing inconclusive results. The use of validated national registries means that our data was not subject to recall bias or differential misclassification of exposures or outcomes. While we could not account for over-the-counter preeclampsia interventions such as low-dose aspirin, no compelling associations between childhood cancer and maternal aspirin intake have been seen in the literature [19, 20].

### Antihypertensive medication use and risk of childhood cancer

Medication is not recommended for mild preeclampsia. For preeclampsia where medication use is indicated, first-line treatments include methyldopa (despite possible hepatic disturbances) and labetalol, noted to have an association with fetal growth restriction [18]. Second-line agents include slow-release nifedipine, and some beta-blockers (other than atenolol). In the US, the prevalence of pregnancy antihypertensive use increased from 3.5 to 4.9% during the years 2000–2007, with the highest use in the first and third trimesters [36]. Definitions and treatment guidelines for hypertensive disorders in pregnancy have not progressed over time the same way those for general hypertension have [18]. The prevalence of overall antihypertensive medication use we observed in Denmark was lower (1.5%) between 1995 and 2014. Diuretics have been a common therapy for chronic hypertension among women of childbearing age, but in 2011 the World Health Organization recommended against their use for preeclampsia-related symptoms due to aggravation of plasma reduction [18]. Despite this, is not clear how frequently diuretics are still given to treat preeclampsia and hypertension in pregnancy. In our sample, diuretics were used by 2.1% of mothers with preeclampsia before 2011, while use increased slightly to 2.8% in 2011–2014.

A study conducted in Germany reported that diuretics and antihypertensives were linked to both ALL and neuroblastoma [19]. While this study was large for a childhood cancer study (N = 639 cases of ALL and N = 158 of neuroblastoma), only 38 mothers of cases took antihypertensive medication, and authors relied on self-report for ascertaining drug use. Although the current study includes 47 mothers of cases who took any antihypertensive medication, drug use is not self-reported. Two other studies which collected data by parental interview reported increased risks for CNS tumor (OR = 2.0) [21] and neuroblastoma (OR = 4.1, 95% CI [1.0–16.9]) [22] amongst offspring of women who took diuretics during pregnancy.

Overall, we observed the strongest associations between preeclampsia and cancer in offspring for births occurring before 1994. Because the Danish Prescription register did not begin until 1995, we cannot determine if diuretic use explained these increased risks, although associations with greater preeclampsia severity would suggest diuretics may have been more likely to have been used. Alternatively, shorter gestational ages in the later time period—suggesting better management of preeclampsia as the study period progressed—would indicate a shorter time period that the fetus was “exposed” to the preeclamptic pregnancy; this may have also decreased the risk of subsequent childhood cancer in the second half of the study period.

Preeclampsia poses a number of risks to the fetus, including growth retardation and complications related to premature delivery [18]. The mechanism by which preeclampsia may increase risk for childhood cancer is unclear. However, neonates born to preeclamptic pregnancies are more likely to be admitted to the neonatal intensive care unit (NICU), with the risk increasing in pregnancies with severe preeclampsia [40]. Prior studies have speculated that treatments incurred in the NICU may increase risk for childhood cancer, including nitric oxide [41], diagnostic radiation, high-fraction oxygen, and parenteral nutrition [9]. Yet, the shorter gestations we observed in the latter study period would suggest a potentially higher risk of infants being admitted to the NICU, and the lower cancer risk in this period would not support that the preeclampsia link with cancer is due to NICU-related interventions.

### Preeclampsia and risk of childhood cancer in offspring

With regards to preeclampsia, we observed excesses of neuroblastoma in subgroup analyses only, e.g., among 1994+ cases, both before and after adjustment for BMI, and after excluding pregnancies with diuretics use. Two previous studies reported no associations between preeclampsia and risk of neuroblastoma [16, 17]. Another study reported an association with “pregnancy-related hypertension” for neuroblastoma cases diagnosed between one and six months and a weaker increased risk for children diagnosed at all ages [37] Neuroblastoma diagnosed at an earlier age may be a different phenotype than neuroblastoma diagnosed after age 1, as shown by studies that contrasted risk factors and survival by age [38]. Neuroblastoma can cause fetuses to be hydropic, which can lead to an abnormal enlargement of the placenta, and in some cases the development of preeclampsia symptoms [39]; however this did not explain our results as none of our neuroblastoma cases had hydrops.

Previous reports on CNS tumors and preeclampsia are limited. One study found no association for CNS tumors overall, but a possible elevated risk for CNS germ cell tumors [8]. With regards to ALL, three studies reported weakly elevated results (OR range 1.1–1.3) for associations with mothers with either preeclampsia or hypertension and one observed a dose–response effect [8, 15, 31], however none of these had information on medication use.

Other possible mechanisms between preeclampsia and childhood cancer risk include the mother’s heightened systemic inflammatory response [42]. Placental dysfunction, an innate characteristic of preeclampsia, has been found to be associated with some childhood cancers [43]. An underdeveloped placenta may allow the fetus to be exposed to higher levels of xenobiotics due to decreased effectiveness as a protective barrier, and is also associated with other negative perinatal outcomes Additionally, altered proinflammatory cytokines and their consequences, including heightened vulnerability for disease, are another possible mechanisms to explain the association between preeclamptic placental dysfunction and childhood cancer risk [44].

## Conclusions

Our results support that diuretic use in pregnancy is a risk factor for childhood cancer. Overall, we observed some associations between preeclampsia and childhood cancer, but more work is needed to determine if this can be explained by diuretic use in pregnancy. These findings highlight the importance of adequate disease management for pregnant women diagnosed with preeclampsia or hypertension, including adherence to medication safety guidelines.

### Supplementary Information

Below is the link to the electronic supplementary material.

Supplementary file1 (DOCX 22 KB)

Supplementary file2 (DOCX 24 KB)

Supplementary file3 (DOCX 22 KB)

## Data Availability

The datasets analyzed in the current study are subject to the General Data Protection Regulation, with restrictions on data sharing in place.
