# Supplementary material for: Preeclampsia, antihypertensive medication use in pregnancy and risk of childhood cancer in offspring
Source: Cancer Causes Control. 2023 Aug 3;35(1):43–53. doi: 10.1007/s10552-023-01745-4 (PMC10764520; doi:10.1007/s10552-023-01745-4)
Supplement: Supplementary file 1 — Supplementary file1 (DOCX 22 KB) [file 10552_2023_1745_MOESM1_ESM.docx]

SUPPLEMENTAL TABLE S1. International Classification of Disease codes used for hypertensive disorders occurring during pregnancy, Anatomical Therapeutic Chemical codes used for antihypertensive medications during pregnancy

|  | Code Used | Classification used |  |
| --- | --- | --- | --- |
|  |  |  |  |
| **ICD^a^ 8** | 637 | Preeclampsia, eclampsia, and toxemia |  |
|  | 637.0x | Preeclampsia |  |
|  | 637.1x | Eclampsia |  |
|  | 637.9x | Toxemia, unspecified |  |
|  |  |  |  |
| **ICD 10** | O14 | Preeclampsia |  |
|  | O14.0x | Mild to moderate preeclampsia |  |
|  | O14.1x | Severe preeclampsia |  |
|  | O14.2x | HELLP syndrome |  |
|  | O14.9x | Unspecified preeclampsia |  |
|  |  |  |  |
|  | O15 | Eclampsia |  |
|  | O15.0x | Eclampsia during pregnancy |  |
|  | O15.1x | Eclampsia during childbirth |  |
|  | O15.2x | Eclampsia during postpartum |  |
|  | O15.9x | Unspecified eclampsia |  |
| **ATC ^b^** | C02 | Antihypertensives |  |
|  | C03 | diuretics |  |
|  | C07 | β-blockers |  |
|  | C08 | Calcium channel blockers |  |
|  | C09 | Agents acting on the renin–angiotensin system |  |

^a^ International Classification of Diseases

^b^ Anatomical Therapeutic Chemical
