# Supplementary material for: Preeclampsia, antihypertensive medication use in pregnancy and risk of childhood cancer in offspring
Source: Cancer Causes Control. 2023 Aug 3;35(1):43–53. doi: 10.1007/s10552-023-01745-4 (PMC10764520; doi:10.1007/s10552-023-01745-4)
Supplement: Supplementary file 2 — Supplementary file2 (DOCX 24 KB) [file 10552_2023_1745_MOESM2_ESM.docx]

SUPPLEMENTAL TABLE S2. Association between maternal preeclampsia during pregnancy and risk for cancer in offspring, Denmark (1977-2013 Sample)

|  |  | **Children whose mother had preeclampsia** | **Crude Model** | **Adjusted Model 1*** |
| --- | --- | --- | --- | --- |
|  | Total N | N (%) | OR (95%CI) | OR (95%CI) |
| Controls | 160484 | 5490 (3.4) | Ref | Ref |
| All cancers | 6420 | 243 (3.8) | 1.10 (0.96, 1.25) | 1.09 (0.95, 1.24) |
| Acute lymphoblastic leukemia | 1222 | 57 (4.7) | 1.41 (1.07, 1.86) | 1.36 (1.03, 1.79) |
| Acute myeloid leukemia | 250 | 10 (4.0) | 1.15 (0.60, 2.19) | 1.15 (0.60, 2.21) |
| Hodgkin lymphoma | 352 | 8 (2.3) | 0.69 (0.34, 1.42) | 0.69 (0.34, 1.42) |
| Non-Hodgkin lymphoma | 163 | 5 (3.1) | 1.17 (0.47, 2.90) | 1.12 (0.45, 2.81) |
| Burkitt lymphoma | 103 | 5 (4.9) | 1.70 (0.67, 4.32) | 1.55 (0.61, 3.96) |
| Central Nervous System tumors | 1583 | 65 (4.1) | 1.19 (0.93, 1.54) | 1.19 (0.92, 1.54) |
| Astrocytoma | 502 | 16 (3.2) | 0.98 (0.59, 1.63) | 0.95 (0.57, 1.59) |
| Intracranial and Intraspinal  Embryonal Tumor | 674 | 21 (3.1) | 0.92 (0.59, 1.43) | 0.91 (0.58, 1.42) |
| Neuroblastoma | 275 | 13 (4.7) | 1.30 (0.73, 2.31) | 1.33 (0.74, 2.36) |
| Retinoblastoma | 140 | 7 (5.0) | 1.55 (0.71, 3.41) | 1.53 (0.69, 3.39) |
| Unilateral | 98 | 5 (5.1) | 1.44 (0.57, 3.65) | 1.39 (0.54, 3.53) |
| Rhabdomyosarcoma | 149 | 5 (3.4) | 1.24 (0.50, 3.08) | 1.22 (0.48, 3.09) |
| Wilms tumor | 203 | 7 (3.4) | 1.03 (0.48, 2.22) | 1.05 (0.49, 2.28) |
| Bone tumors | 270 | 7 (2.6) | 0.64 (0.30, 1.37) | 0.63 (0.29, 1.35) |
| Melanoma | 190 | 7 (3.7) | 1.10 (0.51, 2.39) | 1.15 (0.53, 2.50) |

* Model adjusted for maternal age at delivery, first born child, mother's place of birth, urban or rural deliveries, hypertension before index pregnancy, atopic conditions (lifetime), rheumatoid arthritis before pregnancy, epilepsy (lifetime).
