# Supplementary material for: Preeclampsia, antihypertensive medication use in pregnancy and risk of childhood cancer in offspring
Source: Cancer Causes Control. 2023 Aug 3;35(1):43–53. doi: 10.1007/s10552-023-01745-4 (PMC10764520; doi:10.1007/s10552-023-01745-4)
Supplement: Supplementary file 3 — Supplementary file3 (DOCX 22 KB) [file 10552_2023_1745_MOESM3_ESM.docx]

SUPPLEMENTAL TABLE S3: Diagnosis of either preeclampsia or maternal chronic hypertension during the index pregnancy, and childhood cancer risk

|  |  | Crude Model | Adjusted Model 1 * |  |
| --- | --- | --- | --- | --- |
|  | N (%) | OR (95% CI) | OR (95% CI) |  |
| Controls | 6016 (3.7) | Ref | Ref |  |
| All cancers | 254 (4.0) | 1.09 (0.96, 1.24) | 1.08 (0.95, 1.23) |  |
| Acute lymphoblastic leukemia | 62 (5.1) | 1.42 (1.09, 1.85) | 1.38 (1.06, 1.79) |  |
| Acute myeloid leukemia | 10 (4.0) | 1.07 (0.56, 2.05) | 1.07 (0.56, 2.05) |  |
| Hodgkin lymphoma | 8 (2.3) | 0.62 (0.31, 1.27) | 0.62 (0.30, 1.26) |  |
| Non-Hodgkin lymphoma | 5 (3.1) | 1.04 (0.42, 2.59) | 1.00 (0.40, 2.51) |  |
| Burkitt lymphoma | 5 (4.9) | 1.42 (0.56, 3.59) | 1.31 (0.52, 3.34) |  |
| Central Nervous System tumor | 70 (4.4) | 1.16 (0.91, 1.49) | 1.16 (0.91, 1.48) |  |
|  |  |  |  |  |
| -Astrocytoma | 18 (3.6) | 1.00 (0.61, 1.61) | 0.97 (0.60, 1.58) |  |
| -Intracranial and Intraspinal Embryonal Tumor | 21 (3.1) | 0.82 (0.52, 1.27) | 0.80 (0.51, 1.25) |  |
|  |  |  |  |  |
| Neuroblastoma | 14 (5.1) | 1.29 (0.74, 2.24) | 1.31 (0.75, 2.29) |  |
| Retinoblastoma | 8 (5.7) | 1.62 (0.77, 3.39) | 1.59 (0.75, 3.35) |  |
| -Unilateral | 6 (6.0) | 1.64 (0.70, 3.87) | 1.57 (0.66, 3.72) |  |
| Rhabdomyosarcoma | 7 (4.7) | 1.47 (0.67, 3.19) | 1.55 (0.71, 3.40) |  |
| Wilms Tumor | 7 (3.4) | 0.90 (0.42, 1.93) | 0.90 (0.42, 1.94) |  |
| Bone tumors | 8 (3.0) | 0.69 (0.34, 1.41) | 0.68 (0.33, 1.39) |  |
| Melanoma | 7 (3.7) | 1.01 (0.46, 2.19) | 0.98 (0.44, 2.14) |  |

* Model adjusted for maternal age at delivery, first born child, mother's place of birth,

urban or rural deliveries, atopic conditions during any period, rheumatoid arthritis before

pregnancy, epilepsy during any period.
